# UK validation of the Tinnitus Functional Index (TFI) in a large research population

**DOI:** 10.1186/1745-6215-16-S1-P20

**Published:** 2015-05-29

**Authors:** Kathryn Fackrell, Deborah A Hall, Johanna G Barry, Derek J Hoare

**Affiliations:** 1 NIHR Nottingham Hearing Biomedical Research Unit, Division of Clinical Neuroscience, School of Medicine, University of Nottingham, Nottingham, NG1 5DU, UK; 2 MRC Institute of Hearing Research Clinical Section, Queens Medical Centre, Nottingham, NG7 2UH, UK

## Background

Questionnaires are essential for measurement of tinnitus severity and treatment-related change. Yet no standard measure is used across clinical and research settings. Current tinnitus questionnaires are limited to measuring severity or change, but not both [1,2]. The Tinnitus Functional Index (TFI) was developed as both a diagnostic measure of the functional impact of tinnitus and to be a sensitive measure of treatment-related change [3]. It has not, however, been fully validated. This present study evaluates the validity of the TFI as a diagnostic measure of tinnitus severity in a UK research population.

## Methods

The study involved a retrospective analysis of data collected for 294 participants who had previously participated in a randomised controlled trial of a novel tinnitus device [4]. Participants completed a series of questionnaires including the TFI, Tinnitus Handicap Inventory (THI) [5], Tinnitus Handicap Questionnaire (THQ) [6], a Visual Analogue Scale of Loudness (VAS-L) [7], a Percentage Rating of Annoyance (PR-A), Beck’s Depression Inventory (BDI) [8], Beck’s Anxiety Inventory (BAI) [9], and the World Health Organisation Quality of Life Bref (WHOQOL) [10]. Forty four participants, who completed the TFI at a second visit (within 21 days), provided data for reliability assessment. Psychometric analyses included (1) Confirmatory factor analysis (CFA) using the current eight subscales proposed for the TFI development; (2) Convergent and discriminant validity; and (3) Test-retest reliability and agreement.

## Results

The TFI structure showed acceptable model fit (RMSEA = 0.65), although it was not optimal. The auditory factor in particular was unrelated to the other factors and the underlying construct of “the functional impact of tinnitus on daily life”. Acceptable convergent and discriminant validity was demonstrated by the high correlations between the scores on the TFI and THI (*r* = 0.82) and THQ (*r* = 0.82), moderate correlations with VAS-L (*r* = 0.46), PR-A (*r* = 0.58), BDI (*r* = 0.57), BAI (*r* = 0.38) and WHOQOL (*r* = -0.48). Reliability assessments indicated high test-retest reliability for the TFI global scores (ICC = 0.86) and borderline acceptable test-retest agreement for the TFI global scores (93%).

## Conclusion

The TFI does cover multiple symptom domains and provides a valid measure of tinnitus that can reliably distinguish between individuals in this population. The proposed 8-factor structure, however, was not fully confirmed. Given these results, it may be more appropriate to view each subscale individually rather the combining the subscales to create a global score. Further investigation of the TFI structure and sensitivity to treatment-related change is warranted.
